# Direct Ink Write Printing of Chitin-Based Gel Fibers with Customizable Fibril Alignment, Porosity, and Mechanical Properties for Biomedical Applications

**DOI:** 10.3390/jfb13020083

**Published:** 2022-06-16

**Authors:** Devis Montroni, Takeru Kobayashi, Taige Hao, Derek Lublin, Tomoko Yoshino, David Kisailus

**Affiliations:** 1 Department of Materials Science and Engineering, University of California at Irvine, Irvine, CA 92697, USA or devis.montroni@unibo.it (D.M.); taigeh3@uci.edu (T.H.); 2 Department of Chemistry “G. Ciamician”, Alma Mater Studiorum-University of Bologna, Via Selmi 2, 40126 Bologna, Italy; 3 Department of Biotechnology, Tokyo University of Agriculture and Technology, 2-24-16 Naka-cho, Koganei 184-8588, Tokyo, Japan; s210951u@st.go.tuat.ac.jp (T.K.); y-tomoko@cc.tuat.ac.jp (T.Y.); 4 Materials and Manufacturing Technology Program, School of Engineering, University of California at Irvine, Irvine, CA 92697, USA; dlublin@uci.edu

**Keywords:** polysaccharide, biopolymer, direct ink write printing, additive manufacturing, porous, biocompatible, hydrogel, water content, exposed surface, mechanical properties

## Abstract

A fine control over different dimensional scales is a challenging target for material science since it could grant control over many properties of the final material. In this study, we developed a multivariable additive manufacturing process, direct ink write printing, to control different architectural features from the nano- to the millimeter scale during extrusion. Chitin-based gel fibers with a water content of around 1500% were obtained extruding a polymeric solution of chitin into a counter solvent, water, inducing instant solidification of the material. A certain degree of fibrillar alignment was achieved basing on the shear stress induced by the nozzle. In this study we took into account a single variable, the nozzle’s internal diameter (NID). In fact, a positive correlation between NID, fibril alignment, and mechanical resistance was observed. A negative correlation with NID was observed with porosity, exposed surface, and lightly with water content. No correlation was observed with maximum elongation (~50%), and the scaffold’s excellent biocompatibility, which appeared unaltered. Overall, a single variable allowed a customization of different material features, which could be further tuned, adding control over other aspects of the synthetic process. Moreover, this manufacturing could be potentially applied to any polymer.

## 1. Introduction

Control of architectural features of a material is a crucial aspect in material science [1]. Despite identical composition, different architectures can lead to significant changes in, for example, structural [2,3,4], chemical or physical adsorption [5], or photonic [6,7] properties. These features can be modulated at different length scales [8], from the nano- to the macro-scale, using many different manufacturing processes. However, many of these routes present limitations in scalability, primarily due to economic or technical restrictions. Moreover, the architectural features are usually addressed in a limited dimensional scale. Thus, there is a need to develop scalable manufacturing solutions to provide control over a material’s architecture at different length scales, while still maintaining the ability to integrate multifunctionality, and reduce both cost and environmental impact.

Inducing a shear stress on a solution prior to solidification (usually by solvent elimination) to control the alignment in materials has been widely studied in order to control the material organization at the nano- and micrometric level. Generally, this is obtained by extruding a material through a needle yielding a macroscopic material (either a 1D fiber or a 3D organized material, if combined with 3D printing). This approach has been used on different materials, such as nanocrystals or polymers (both synthetic and biogenic) solutions, to produce matrices with high fibrillar alignment along the extrusion direction [9,10]. Raney et al. even reported the inducing of an angle of the fibril alignment by applying a spinning motion to the nozzle [11]. Moreover, Noroozzi et al. (2021) showed how, in 3D printing, the geometry of the printed material also strongly influenced the final material properties, adding an additional level to the hierarchical structure of the material [12].

Using this approach, hydrated materials can also be printed by using swellable polymers, such as alginate or agarose, by rapid crosslinking of highly viscous solutions [13,14], or adding to the mixture a self-assembling mineral phase that acts as a support [15]. In order to obtain a gel-like material, the conditions need to be specifically optimized based on the chemistry of the components. A general approach to obtain highly swelled materials from polymers has not been reported.

Most extrusion processes target synthetic polymers, due to their easy handling, specific chemical composition and size (i.e., average molecular weight). Conversely, biopolymers are generally less utilized. Cellulosic (usually nanocrystals) or collagen-based materials have been prepared using this approach, exploiting their biocompatibility for medical applications [16]. These studies frequently use a mixture of synthetic and bio-polymers, or chemically-modified biopolymers to allow for UV curing or other specific interactions leading to cross-linking [15,17]. Pure biopolymers have rarely been utilized as the only material or major component of the mixture. Amongst these natural polymers, chitin has scarcely been investigated as the primary print media and has never been used as single component.

In this study, we developed an additive manufacturing process, direct ink write printing, to control different architectural features from the nano- to the millimeter scale (i.e., diameter and fibrillar alignment) during extrusion to obtain chitin-based gel scaffolds. The approach we utilize can potentially be expanded to any polymeric material to obtain materials with a single hydrated component. Here, a single variable has been addressed, but future studies will focus on a multivariable analysis of this system. Chitin was chosen as the polymer to test this system as this biopolymer grants different advantages since it is cheap, mechanically robust [18,19,20], and has a propensity to self-assemble into crystalline nano-fibrils [21,22,23]. Moreover, the biocompatibility of this polymer promotes its use as material for medical applications, from drug delivery to wound healing and regenerative medicine [24,25,26,27,28,29]. For this reason, the scaffolds obtained were tested to evaluate how they interact with cells in vitro.

## 2. Materials and Methods

### 2.1. Chitin Gel Fibers Preparation

A 1 wt./vol.% chitin solution (5–10 mL) was prepared using α-chitin from crab shell dissolved in 1,1,1,3,3,3-hexafluoro-2-isopropanol (HFIP) in a glass vial and stirred overnight. Both reagents were purchased from Sigma Aldrich (St. Louis, MO, USA). The same batch of chitin was used during the whole data collection.

The chitin solution was loaded in a syringe and was allowed to rest for 10 min to eliminate trapped air bubbles. A steel nozzle was then mounted on the end of the syringe and the system was placed in a BIO X, CELLINK 3D printer (Cellink LLC, Boston, MA, USA). The tip of the nozzle was suspended in a 50 mL plastic vial filled with distilled water, with the nozzle tip immersed within the water. Different gauge cylindrical steel nozzles were tested: 20G (0.81 mm nozzle internal diameter, NID), 22G (NID 0.64 mm), 25G (NID 0.455 mm) and 27G (NID 0.361 mm), while only one plastic conical nozzle was tested, 22G (NID 0.41 mm). All nozzles were purchased from CELLINK. The solution was then extruded using a controlled pressure of 75 kPa to obtain a continuous gel fiber with desired length. After printing, the gel fiber was left in DI water overnight to equilibrate and allow the organic solvent to diffuse out of the fiber prior to usage.

The samples used for biocompatibility tests were prepared by extruding the solution into sterile water and using a plastic vial sterilized with 70 vol.% ethanol.

### 2.2. Wet Diameter and Water Content Measurement

The fiber’s wet diameter was measured by laying a fiber on a glass slide, eventually eliminating the excess of water by blotting with a tissue (i.e., Kimwipe). At least 6 different locations were imaged along a >3 cm long fiber. The images were collected using an AmScope SM-2T optical microscope (AmScope, Irvine, CA, USA) equipped with an AmScope MU500 camera at 4.5× magnification. Image analyses were performed using ImageJ [30,31]. The error of the measurement was determined as the standard deviation on the >6 measurements performed.

The water content of the gel fibers was measured by weighing a wet fiber lightly blotted on a tissue. The fiber was subsequently left overnight at room temperature in a desiccator under vacuum and then re-weighed. Each sample measurement was repeated at least twice on independent samples. A Quintix35-1S Sartorius balance was used to determine the fiber weight. The water content was calculated as: water% = (wet wt./dry wt.) × 100. The error of the measurements were determined as the standard deviation on the results of 2 or 3 independent samples.

### 2.3. SEM and Optical Microscope Imaging

Optical micrographs were collected on wet samples laid on glass slides, eventually eliminating the excess of water with a tissue. The images were acquired using an AmScope SM-2T optical microscope equipped with an AmScope MU500 camera and using 4.5× magnification.

Each specimen was dehydrated prior to conducting scanning electron microscopy (SEM) imaging using an ethanol gradient (from 0 vol.% to 90 vol.%, in 10 vol.% increments of 1 h at 4 °C, then 95 vol.%, 100 vol.% and 100 vol.% for 1 h at 4 °C). Finally, the remaining ethanol was eliminated by critical point drying using CO_2_. The specimen was mounted on carbon tape attached to an aluminum stub. The specimen was handled with care, by picking up only one of the ends of the fiber and laid across the carbon tape, in order to avoid mechanical damage from the forceps. Each sample was coated with 5 nm of Ir prior to imaging and imaged using a FEI Magellan 400 XHR SEM (FEI company, Hillsboro, CA, USA) at 3 kV and 13 pA.

### 2.4. Image Analyses

Collected SEM micrographs were analyzed using OrientationJ (Version 2.0.5, National Institutes of Health, Bethesda, MD, USA), a plugin of ImageJ. For each specimen, four different parameters were calculated in duplicate (on two independent SEM samples) using images acquired at 25,000× magnification. Micrographs imaged at 50,000× were used if there was more than one preferential direction or if too flat of a distribution was observed. The fibril-fiber coherence was determined as the absolute value of the difference between the angular direction of the fiber (image at 350×) and the calculated angular direction of fibril distribution. The distribution of orientation represents the difference of distribution between the maximum and its orthogonal direction. The full width at half maximum was calculated by normalizing the distribution to zero. Finally, the percentage of coherency was calculated for the dominant direction. A schematic of these calculations is reported in Figure S1 in the Supplementary Materials. The error associated with each result was calculated as a standard deviation of the analyses of the two images analyzed, for each sample.

### 2.5. Uniaxial Tensite Tets

Uniaxial tensile tests were performed using a Bose Electroforce 3200 Dynamic Mechanical Tester equipped with a 50 g loadcell manufactured by Honeywell (TA Instruments, New Castle, DE, USA). A wet fiber was mounted on a plastic frame (with a ~1.8 cm window) using superglue to fix the fiber’s edge to the frame, and sodium bicarbonate was used as an accelerant to achieve glue curing in few seconds. The actual length of the fiber was measured with a caliper (±0.01 mm). The frame was mounted on the instrument, then the sides of the frame were cut to release the fiber for testing. The tests were conducted using a strain rate of 0.05 mm·s^−1^ for all samples. A humidifier was used to keep fibers fully hydrated before and during testing. The average wet diameter previously measured was used to calculate the stress. The Young’s modulus was measured as a linear interpolation of the strain range between 5% and 30%. At least 6 independent samples were tested for each condition, and the error associated with the result was obtained as a standard deviation.

### 2.6. Biocompatibility Tests

Cytotoxicity assay of the chitin gel fibers was performed using a Cell Counting Kit-8 (CCK-8), which allows sensitive colorimetric assay for the cell viability. MCF-7 human breast cancer cells (ATCC, Manassas, VA, USA), which are the most studied human adherent cells, were cultured in Eagle’s minimal essential medium (MP Bio, Santa Ana, CA, USA) supplemented with 1 mM sodium pyruvate, 10 µg/mL insulin, 1.5 mg/mL sodium bicarbonate, 10 vol.% fetal bovine serum, and 1 vol.% penicillin-streptomycin. Cultures were maintained at 37 °C and 5% CO_2_ for approximately 1 week. Prior to each experiment, confluent cell cultures were washed with phosphate-buffered saline (PBS, pH 7.4) followed by trypsinization and resuspension in PBS. The cells were seeded into 48-well at 2.5 × 10^4^ cells·well^−1^ in a total volume of 200 μL. The gel fibers (22G, and 27G), cut into 1 cm pieces, were treated with boiling water for 5 min and then introduced into the wells. The cells with fiber gels were cultured at 37 °C and 5% CO_2_ for 24–144 h (1–6 days). A CCK-8 solution (20 μL) was added into the each well at 0, 24, 48, 72, 96, 120, and 144 h followed by color reaction. After incubation for 4 h, the absorbance at 450 nm was measured. The condition of the cells around the fiber gels was observed under an inverted microscope (OLYMPUS IX71, Olympus Corporation, Tokyo, Japan). All results were performed in triplicate.

## 3. Results

### 3.1. Nano-Organized Chitin-Based Gel Fibers’ Synthesis and Characterization

**Figure 1:**
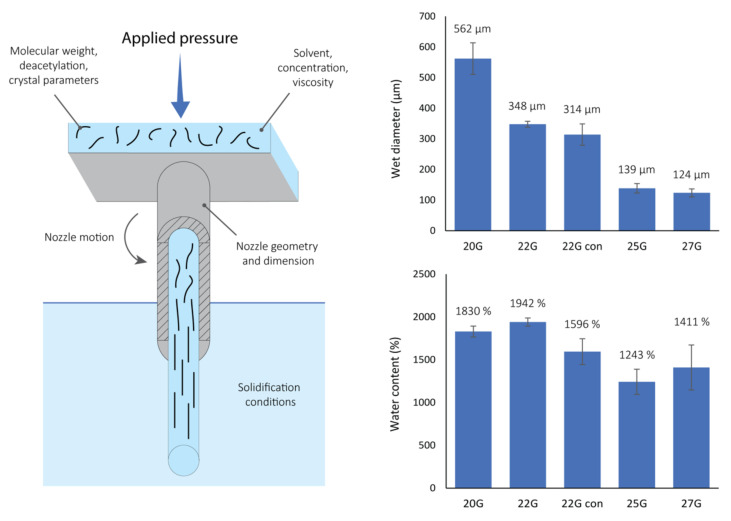
On the (**left**), a schematic representation of the extrusion setup showing all the possible control variables. In this study, the general nozzle size and geometry were examined. The (**upper right**) shows the wet diameters for the gel fibers obtained using the different nozzles (number of measures per condition, N ≥ 6), while the (**lower right**) depicts the percent water content measured for gel fibers printed with the different nozzles (number of samples per condition, N ≥ 2). For each condition the average value is reported above each histogram column.

The chitin-based hydrogel fibers utilized in this study have been synthesized starting from a homogenous 1 wt./vol.% solution of α-chitin in HFIP. The synthesis was performed via a controlled extrusion at 75 kPa of the solution into a counter-solvent (Figure 1), in this case distilled water, that induced the precipitation of the biopolymer. A screening study of the nozzle internal diameter (NID) was performed to determine its influence in the resulting fiber diameter and molecular alignment in the chitin-based hydrogel fibers. Four steel cylindrical nozzles were tested: 20G (NID 0.81 mm), 22G (NID 0.64 mm), 25G (NID 0.455 mm), and 27G (NID 0.361 mm). In addition, a 22G plastic conical nozzle, 22G con, (NID 0.41 mm), was tested.

**Figure 2:**
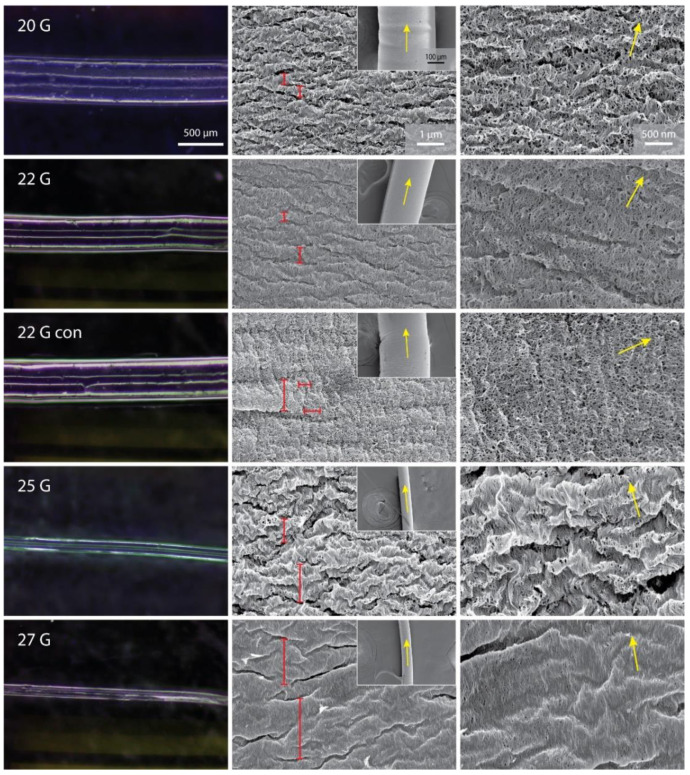
Optical micrographs of the wet gel fibers (first column), and SEM micrographs showing the entire fiber width at low magnification (inset, second column), the surface at intermediate magnification (second column), and the relative chitin fibril alignment at high magnification (third column) of the samples obtained using the different nozzles. Yellow arrows show the fiber extrusion direction (second column, inset) and the calculated chitin fibril alignment direction (third column). Red segments in the central column highlight distances between wrinkles in printed fibers.

All printed chitin hydrogel fibers were transparent gel fibers (as can be observed in the optical microscopy images, Figure 2), barely visible in water. The length of the fibers is controlled by the duration of the extrusion process, potentially leading to meter-long fibers. For example, scaffolds more than 0.5 m long have been easily obtained, suggesting the scalable nature of this process that is only limited by the size of the syringe and immersion tank. The wet diameters of the fibers were dependent on the nozzle used. Figure 1 highlights the lower limits (in this study) of fiber diameters using nozzles with a lower NID. Comparing the fiber diameter to the NID, the cylindrical nozzles showed a contraction of the extruded material of about 250–300 µm. A contraction of about 100 µm was observed for the conical nozzle.

The water content of the different fibers, reported in Figure 1, was between 1200 and 2000 wt.% (i.e., only between 8.3 and 5.0 wt.% of the scaffold was chitin). Considering the standard deviation, the scaffolds showed comparable water content. A general decrease trending with lower NIDs was observed, with the largest observed water content for the 22G nozzle and the smallest for the 25G nozzle.

The TGA analysis of the fibers (Figure S2) confirmed that most of the fiber weight was made up by water. Generally, the lower the NID used to produce the fiber, the lower temperature was observed to be required to fully dehydrate the scaffold, except for sample “22G con” which exhibited the highest temperature (comparable to 20G) to fully dehydrate the matrix. An analogue trend was observed in the heat flow associated to the dehydration event where a more negative heat flow was associated to bigger NID, while “22G con” showed the most negative heat flow peak value.

### 3.2. Micro- and Nano-Structural Analyses of the Chitin Gel Fibers

In order to evaluate the micro- and nano-structural features of the scaffold, the samples were dehydrated using an ethanol gradient and subsequently critically point dried prior to analysis using SEM (using a 5 nm Ir coating). As reported in Figure 2, the analyses showed an overall homogenous fiber with semi-periodic (ca. micron scale) wrinkles, orthogonal to the primary fiber direction, on the surface. A fibrillar morphology was observed at the nanoscale level. Among the cylindrical nozzles, there is a general increase in the fibrillar alignment, longitudinal to the primary fiber direction, as a function of decreasing NID. Conversely, an increase of the interspacing of wrinkles was associated with a decrease in NID, leading to larger sized wrinkles associated with a higher fibrillar alignment and vice versa. Interestingly, almost no fibrillar alignment were observed when the conical nozzle was used and the very small wrinkles observed appeared almost parallel to the primary fiber direction. Occasional aberrations or imperfections, such as air bubbles imprinted on the surface were observed.

SEM image analyses of the different scaffolds revealed the effect the cylindrical nozzles had on the degree of fibrillar alignment within each fiber. Specifically, a higher alignment, both in terms of distribution and intensity, was observed using a smaller NID (Table 1). This trend was observed in all the parameters studied. A general fair coherence between the extrusion direction of the fiber and the fibrillar alignment was observed (fibril-fiber coherence), except for the “22G con” nozzle. In this sample, two orientation directions: (i) approximately 90° from one another or (ii) a broad bell-shape with a flat plateau, were observed at 25,000× magnification. The alignment distribution became more unidirectional when analyzing micrographs at 50,000× magnification. This second analysis showed a poor fibril-fiber coherence compared to the cylindrical nozzles. For this reason, no FWHM and coherency were calculated on this sample. A similar flat bell-shape was observed on the 25G samples. The distribution became more oriented using images acquired at 50,000× magnification, obtaining results analogous to the other specimens.

### 3.3. Uniaxial Tensile Tests of the Wet Chitin Gel Fibers

**Figure 3:**
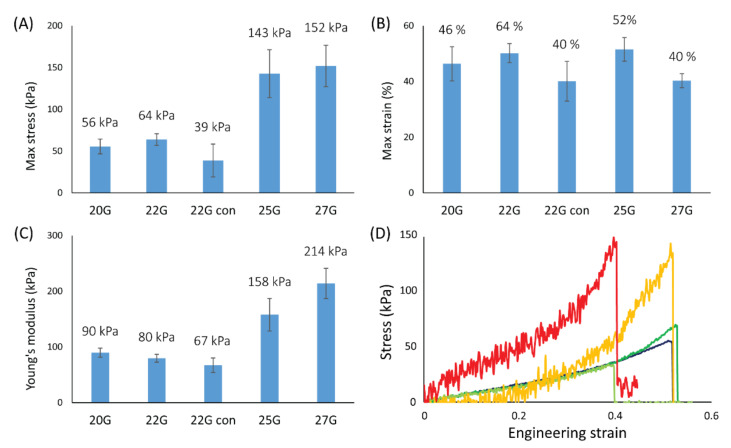
Results of the mechanical tensile tests on the wet fibers. Different mechanical parameters are reported for each of the printing conditions: (**A**) max stress; (**B**) max strain; (**C**) Young’s modulus. Number of samples per condition, N ≥ 6. For each condition the average value is reported above each histogram column. In (**D**) representative stress-strain profiles of the gel fibers studied are reported: 20G (blue), 22G (dark green), “22G con” (light green), 25G (yellow), and 27G (red).

The wet samples were also tested using uniaxial tensile tests to evaluate their mechanical properties, with the results summarized in Figure 3. The stress-strain curves showed an analogous trend for all the samples examined. Each tensile profile showed an initial lower slope (up to about 30% the maximum elongation) that transitions into a higher slope until fiber breakage. Each fiber broke in a single event, and no defibrillation was observed. The three mechanical parameters examined were the maximum stress (σ_max_), maximum strain (ε_max_), and the Young’s modulus (YM). YM was calculated on the first slope observed (between 5% and 30% strain). For each parameter, a t-test was performed on the data belonging to each data set. All samples showed no significant differences in the ε_max_ (ν ≥ 9, *p* = 0.05), showing a value of about 50%. A slightly lower value (40%) was observed for “20G con” and 27G. The σ_max_ (ν ≥ 10, *p* = 0.05) and YM (ν ≥ 11, *p* = 0.05) showed no significant differences for 20G, 22G, and “22G con”. A σ_max_ value of ~60 kPa, and a YM value of ~80 kPa were observed. A significantly higher value was observed for 25G (σ_max_ ~140 kPa, and YM ~160 kPa), and 27G (σ_max_ ~150 kPa and YM ~200 kPa), but no significant difference between these two samples was observed. Aside from the statistical evaluation, increasing trends in both σ_max_ and YM are observable moving from 20G, 22G, 25G and 27G. 

### 3.4. Scaffolds’ Biocompatibility

**Figure 4:**
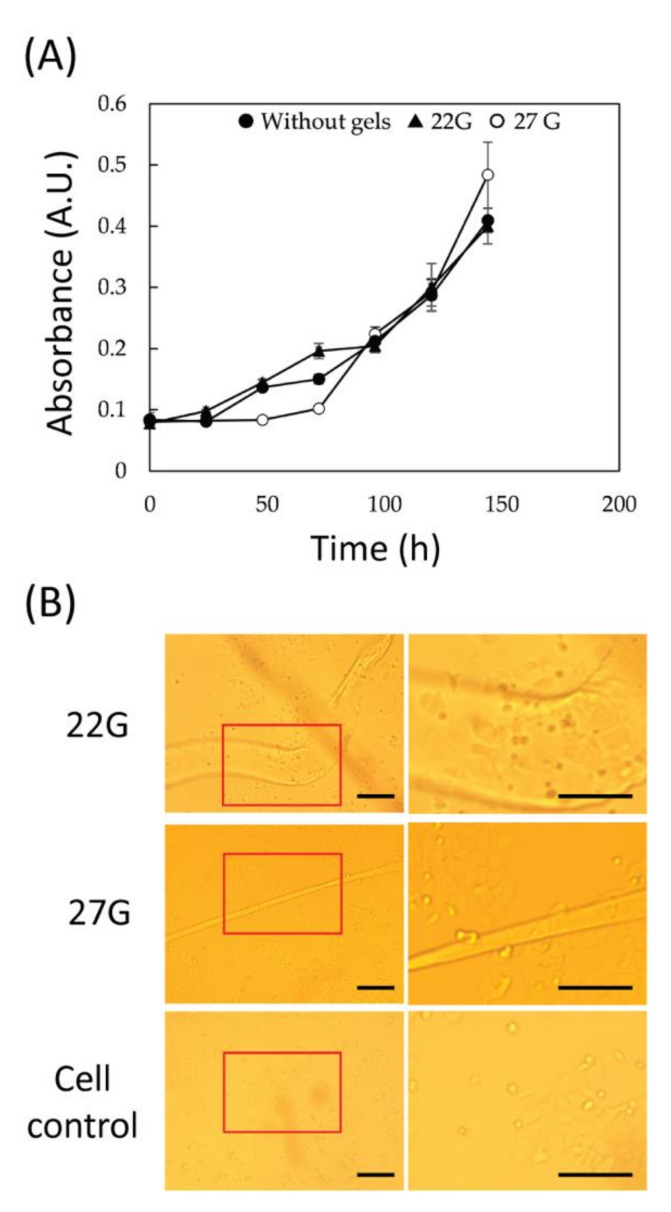
Evaluation of the effect of the fiber gels (22G and 27G) on cell cultures. (**A**) Cell proliferation determined using the Cell Counting Kit-8(CCK8) viability assay (number of samples per condition, N = 3). (**B**) Optical microscope images of MCF-7 cells grown in the presence or absence of the gels in a 48 well plate for 144 h. For each condition, a lower magnification image (**left**) is reported, with a red section highlighting where the higher magnification images (**right**) were taken. Scale bars: 200 µm.

In order to evaluate the biocompatibility of the fibers, a cell culture was performed in presence of the fibers. In this experiment, two types of scaffolds were tested: 22G and 27G, using the same fiber length. The gels were introduced into each well and incubated for 144 h (6 days). Figure 4A shows the cell viability during the culture period. Regardless of the diameter of the gels, there was no clear difference from the culture in the absence of gel. In addition, at the bottom of the well after 144 h from the start of co-culture, it was confirmed that cells were growing around the gels with no difference in density compared to elsewhere in the well (Figure 4B). 

In order to increase the cell adhesion to the scaffold, an introduction of adsorbed collagen within fibers was performed. After this treatment, cells were observed to adhere to the scaffold (see Figure S3).

## 4. Discussion

In this study, a system for the synthesis of a transparent nano-structured biopolymeric gel fibers with customizable water content, mechanical properties, and fibrillar alignment is presented. One of the possible variables of this system was studied to evaluate its influence on the final scaffold architecture. The effect of NID is discussed, which was selected based on facile control and standardization, while granting a strong influence on the micro- and nanoarchitectures and final matrix properties. As an additional test, a nozzle with a conical shape, as opposed to cylindrical, was also utilized to evaluate the influence of geometry.

A screening on the influence of the NID was conducted using one conical and four cylindrical nozzles, at a constant applied pressure of 75 kPa. The starting solution used was 1 wt./vol.% chitin in HFIP. This concentration was chosen as the highest concentration achievable to obtain a homogenous solution of chitin in HFIP using magnetic stirring (we expect more energetic stirring methods will allow access to more concentrated solutions in the future). A high concentration was preferred since it presents a viscosity compatible with extrusion processes and, thus, the possibility to obtain a self-standing gel. The batch of chitin used was the same in the whole study, so no modification in composition should be present. Unfortunately the average molecular weight of the chitin used was not available. This is quite common for chitin, contrary to chitosan, which does not undergo any harsh extraction treatment and is usually sold as a high molecular weight product (few hundreds of kDa) [20]. Since this work represents an initial screening of this system, both concentration and molecular weight were not modified in this study and will be examined in detail in future dedicated works. We expect them to be crucial parameters in determining the mechanical resistance of the gel. For example, increasing the gelifying agent concentration was observed to have a positive correlation with the mechanical resistance [32].

The five matrices obtained from the different nozzles tested were esthetically similar, long transparent gel fibers. This suggests that the precipitation of the biopolymer in the counter solvent was fast enough to inhibit a complete disassembly of the material, and thus resulted in significant fibril alignment and water content. Similar extrusion processes were performed on biopolymers without achieving an analogue fibrillar alignment [10,15]. On the other hand, this gel state is not surprising considering the high hydration that biogenic and synthetic chitin matrices exhibit [33,34,35], which suggests a strong tendency of the polymer to entrap water. Despite this, a shrinkage of the gel was observed overnight after printing. In fact, immediately after extrusion, the fibers had diameters comparable to the NID. However, after soaking overnight in water, a decrease of the gel diameter was observed. This change in diameter may be related to either a local rearrangement/alignment of the bio-polymer fibrils in a more compact conformation (potentially due to more extensive interpolymeric hydrogen bonding, leading to higher crystallinity, or to an increase of the interaction between chitin’s apolar regions during the exchange of water for HFIP) and/or an effect of the osmotic pressure due, again, to the exchange of water for HFIP. It seems that all fibers experienced a similar decrease in diameter, regardless of the nozzle diameter used. The only significant difference was observed for the 22G conical nozzle, which demonstrated an approximately 50% decrease in diameter. If an osmotic pressure were responsible of this shrinkage, we would expect thicker fibers to show a higher decrease in diameter. In fact, the diffusion of the two solvents would require a longer time to reach the fiber bulk. This would imply that fibers obtained from nozzles with similar NID, such as the 25G and the 22G conical nozzles (NID of 0.45 mm and 0.41 mm, respectively) would undergo similar diffusion processes. This would lead to similar final wet diameters, which is opposed to the evidence collected. A major difference observed in the conical nozzle is the significant absence of fibril alignment, while all cylindrical nozzles showed measurable orientation. This, combined with the observed reduction in the decrease of the fiber diameter, suggests that a major factor is the localized reorganization/alignment of the fibrils. The fibrils might in fact have a higher tendency to contract when fibrillar alignment is present, likely to be due to extensive interpolymeric interactions. In fact, a more compact surface (i.e., closer packing of fibrils) is observed in the samples with an overall higher fibrillar alignment. Coherently, Nickien et al. observed how collagen fibrils with a lower fibrillar alignment and lateral interconnectivity showed a swelling about four times higher [36]. This suggests a tendency of aligned fibrils to interact less with the solvent.

The rate of precipitation and the successive fibril compaction may also have an effect on the final water content of the gel. Generally, no strong differences were observed among the samples. However, a general trend shows that thinner nozzles yield fibers with lower water content. As for the wet diameter, a higher water content was observed in fibers from the 22G conical nozzle compared to the fibers printed with the 25G nozzle. These observations suggest that more compact fibrils allow less water to get entrapped in the scaffold. Since a similar shrinkage of the fiber was observed, unrelated to the NID, the fibril compaction event may not be strongly involved in the decrease of the hydration. A possible explanation may be the decrease of the polymer exposed surface, now involved in fibril lateral interactions, with no more ability to interact with water. Similarly, Pajchel and Kolodziejski (2013) observed an increase in hydration, increasing the exposed surface of nano-crystalline calcium hydroxyapatites, by dry milling [37]. In our study, this observation was also supported by the TGA analyses. Among the cylindrical nozzles, a general correlation was observed between the NID and the temperature at which full dehydration occurs in the matrix. This may possibly be related to faster dehydration kinetics due to the smaller fiber diameter. Despite that, we expect more strongly bounded water to be retained in the structure up to higher temperatures (usually above 100 °C) and a requirement for more energy to be evaporated [38]. These water molecules are in fact those in direct contact with the fibrils and are directly linked by hydrogen bonding. This would explain why fibers exhibiting higher fibrillar alignment required a higher temperature to fully dehydrate with more energy (see heat flow analysis) to evaporate water above 100 °C. In fact, the two samples with the highest fibrillar alignment (25G and 27G) were the only ones showing full dehydration before 100 °C, associated with the lowest heat flow. Coherently, the conical nozzle, which exhibited the lower fibrillar alignment (but a wet diameter comparable to the 22G nozzle), showed the highest temperature of full dehydration (comparable to that of the 20G sample). The combination of the swelling, TGA, and wet diameter results support the idea that a lower fibrillar alignment is associated with a lower hydration, since a lower interaction with the solvent is present due to an increase in inter-fibrillar interactions in spite of fibril-solvent interactions.

As previously mentioned, the SEM observation of the dried fibers showed a general increase in fibrillar alignment with smaller NID nozzles. This qualitative observation was confirmed from the image analyses performed. This analysis showed proportionally more intense and sharper bell distributions of fibril orientation when using thinner nozzles. This is coherent with a higher shear stress between the nozzle’s internal wall and the solution during the extrusion process. Thus, this stress would consequently induce alignment in the solution prior to precipitation [39]. Coherently, the conical geometry produced a lower shear stress and induced almost no fibrillar alignment along the extrusion direction [40]. As mentioned, another feature observed with a strong correlation with the fibrillar alignment was the surface porosity and consequent exposed surface.

The cylindrical nozzles showed a fibril-fiber coherency below 16° (i.e., there was no more than 16° of angular divergence between the fibril orientation and the extrusion direction). This represents an almost complete alignment with the extrusion direction, considering a mild deviation in the analyses. Surprisingly, a mildly intense alignment was observed at about 60° from the extrusion direction in the “22G con” specimens. This difference may arise from lateral forces applied on the solution due to the conical shape of the nozzle, before precipitation. These forces may have induced a mild variable vortical motion of the solution close to the nozzle tip, disrupting and modifying the solution fibril alignment and inducing a non-unidirectional orientation. This effect may be exploited in future studies to induce an alignment not along the extrusion direction, potentially coupling the conical nozzle with a rotational motion to get a finer adjustment [11].

At the microscopic level, a semi-periodically spaced wrinkled surface was observed. This morphology may be an artifact due to the dehydration of the samples and may not be present in the native wet scaffold. A qualitative correlation between the dimension of these wrinkles and the NID was observed, with larger wrinkles associated with a lower NID. This effect may arise from two possible effects: (i) a stop-slip effect during the printing due to partial solidification of the polymer at the edge of the nozzle tip, lightly blocking it, and leading to successive expulsion of the material by a local pressure increment; (ii) a higher flexural mechanical resistance of the surface due to increased fibrillar alignment and compaction, which would result in an impediment for the surface to fold into wrinkles. These wrinkles would also contribute to the alignment distribution in the image, sometimes inducing a significant broadening of the bell of distribution (i.e., 25G specimen). For these cases, this contribution was limited by focusing on a smaller area where a reduced number of wrinkles is considered, highlighting the fibrillar alignment. Again, a difference was observed using the conical nozzle. This sample exhibited almost no transversal wrinkles, but instead showed less obvious ones parallel to the long axis of the fiber. This difference may arise from the conical shape of the nozzle, which would laterally compress the solution at the tip, where the solidification process starts to occur, inducing a folding of the fiber surface. Alternatively, it may be due to the different fibrillar orientation in this sample, which would shrink differently during dehydration.

The stress-strain profiles of the fibers under uniaxial tensile tests showed two different slopes, one in the initial range of the curve (up to 30% of strain) and a successively higher one until fiber breakage. This profile may be due to an initial contraction of the fiber diameter with water being extruded from the fiber followed by the actual displacement or stretching of the fibrils. A positive correlation between the fibrillar alignment in the fibers and their σ_max_ and YM was observed. This is not surprising since it is well known how aligned fibrils can positively contribute to the mechanical resistance of a matrix in that specific direction [41]. Coherently, about one third of the σ_max_ and YM values were observed for “22G con” compared to the 25G sample, where two significant degrees of alignment were observed despite starting with a similar NID. This correlation shows how the final material resistance could be easily tuned by controlling the nanostructure of the fiber. Interestingly, no significant differences were observed in the ε_max_, meaning the fibrillar alignment may not be responsible for controlling the deformation of the material. Despite that, fibers appeared quite elastic, showing a deformation of about 50%.

The 20G and 27G fibers were co-cultured with mammalian cells to evaluate the biocompatibility of the material. These fibers were chosen since they represent the two extremes in wet diameter and NID. Although the fiber manufacturing process was expected to introduce substances (i.e., HFIP) that inhibit cell growth, there was no effect on cell growth regardless of fiber size. Some cells were observed to localize in the vicinity of the material, suggesting that modification of the surface of the material promotes cell adhesion and can be used as a scaffold. These results demonstrate chitin’s full biocompatibility, a result that could be expected. In fact, many studies have been using chitin scaffolds in tissue engineering, exploiting its biocompatibility, bio-sorbibility, and atoxicity. Despite this, chitin is known not to induce a strong cell adhesion [29]. For this reason, different methods have been developed to increase cell adhesion. Among these methods, collagen adsorption or coating is one of the most common. A first test of collagen adsorption was performed exposing the wet fiber to a collagen-rich solution for 24 h. The final matrix showed a significant increase of cell adhesion suggesting that this matrix could be further customizable after the synthesis.

The results collected in this study show that the chitin gel fibers obtainable with this novel methodology are easily customizable in dimension, fibrillar alignment, microarchitecture, and mechanical resistance while maintaining similar swelling, biocompatibility, and maximum strain. This customization was demonstrated to be easily achievable by simply using different NID during the extrusion. 

Although this study only targets one variable, the system allows for a strong control over many different parameters (a few of them are reported in the schematic in Figure 1). These parameters may be classified as initial solution conditions, physical factors, and precipitation constraints. Initial solution conditions include those related to the solvent (polarity, hydrogen bonding capability, etc.), the polymer material (molecular weight, crystallinity, etc.), and the solution preparation (concentration, viscosity, etc.). Physical factors include those related to the extrusion process, nozzle used (material, NID, shape, etc.), pressure, and eventual motion of the system (i.e., vibration or rotation of the nozzle). Finally, precipitation constraints include the counter solvent used (polarity, density, miscibility with the initial solvent, etc.) and its physical state (temperature, pressure, etc.). All of these different parameters could enable fine control of the material preparation, such as the shear stress applied to the solution, the rate of precipitation, or the solvent diffusion to, or from, the matrix during the precipitation. Finally, these control parameters could be expressed in a single-step additive manufacturing process that can potentially be expanded to any polymer. This technology could also be potentially translated into a 3D printing technology allowing the production of 3D gel structures with specific properties. In addition, these fibers by themselves, or in 3D structures, could easily find application in biomedical fields such as in regenerative medicine, drug delivery, or the development of grafts or implantable devices.

## 5. Conclusions

In this study, a novel one-step multivariable additive manufacturing process for polymeric gel fiber production with control over its micro- and nano-architectural features is demonstrated. In this initial study, chitin was tested as the polymer and water as a counter solvent, in order to obtain a green and biocompatible final scaffold. A single variable, the NID, was studied to evaluate its influence on the final material properties. Beyond this, future multivariable in-depth studies will be necessary to achieve full control over the manufacturing system capability. The screening of NID influence on the scaffold production shows how a single variable is able to influence many different architectural features. In fact, a positive correlation between NID, fibril alignment, and mechanical resistance was observed, likely to be correlated to the shear stress produced in the nozzle. A negative correlation, still related to the degree of fibril alignment, was observed with porosity, exposed surface, and lightly with water content. No correlation was observed with maximum elongation, and biocompatibility, which always appeared unaltered. Overall, a single variable allowed a customization of many different material features, which could be further tuned, adding control over other aspects of the synthetic process. This would reflect in the production of diversified materials which could find use in many different fields, including biomedical applications. 

## 6. Patents

A provisional disclosure has been filed based on this work: Additive manufacturing of tunable polymeric 3D architectures for multifunctional applications (UC Case No.: 2022-708-1). 

## Figures and Tables

**Table 1:** Fibril alignment parameters obtained from SEM images (25,000×).

NID	Fibril-Fiber Coherence (°)	Distribution of Orientation (10^3^)	FWHM (°)	Coherency (%)
20G	16 ± 1	5 ± 3	90 ± 30	0.03 ± 0.02
22G	16 ± 3	14 ± 4	90 ± 30	0.13 ± 0.04
22G con	60 ^1^ ± 10	13 ^1^ ± 5	n.d.	n.d.
25G	10 ^1^ ± 10	17 ^1^ ± 7	60 ^1^ ± 20	0.14 ^1^ ± 0.02
27G	7 ± 8	37 ± 2	34 ± 1	0.33 ± 0.03

^1^ These data were acquired on images at 50,000× magnification.

## Data Availability

All Data generated or analyzed during this study are included in this published article (and its supplementary information files).

## Supplementary Materials

The following supporting information can be downloaded at: https://www.mdpi.com/article/10.3390/jfb13020083/s1, Figure S1: Schematic of the image analyses performed; Figure S2: TGA analysis of the chitin gel fibers; Figure S3: Confocal images of cells adhering to the chitinous scaffold.
