# Supplementary material for: Direct Ink Write Printing of Chitin-Based Gel Fibers with Customizable Fibril Alignment, Porosity, and Mechanical Properties for Biomedical Applications
Source: J Funct Biomater. 2022 Jun 16;13(2):83. doi: 10.3390/jfb13020083 (PMC9225658; doi:10.3390/jfb13020083)
Supplement: Supplementary file 1 [file jfb-13-00083-s001.zip › jfb-1730180-supplementary.pdf]

Direct ink write printing of chitin-based gel fibers with customizable fibril alignment, porosity, and mechanical properties for biomedical applications

*Devis Montroni, Takeru Kobayashi, Taige Hao, Tomoko Yoshino and David Kisailus **

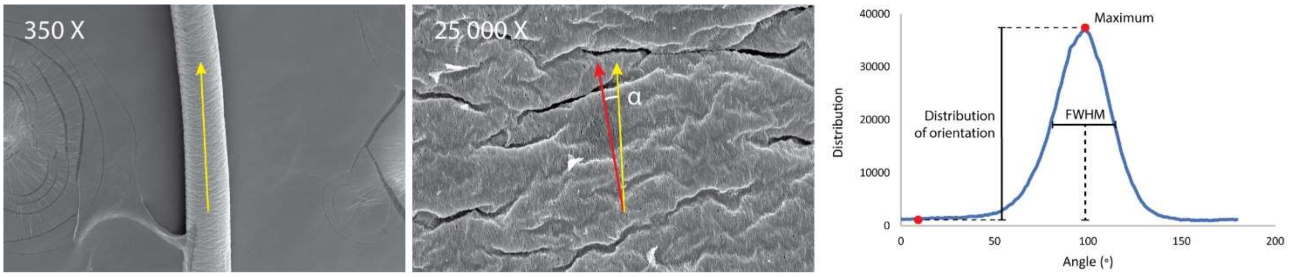

Figure S1: Schematic of the image analyses performed. SEM micrograph of a fiber collected at 350 X, on the left, and 25 000 X, in the center. On the right, the distribution of orientation calculated from the 25 000 X micrograph is reported. Yellow arrows indicate the orientation calculated for the fiber, a red arrow shows the direction of the maximum orientation calculated, the angle α represent the fibril-fiber coherence. In the distribution graph two red dots identify the maximum of orientation of the fibrils and its orthogonal orientation. The segments report graphically how the distribution of orientation and the FWHM were calculated.

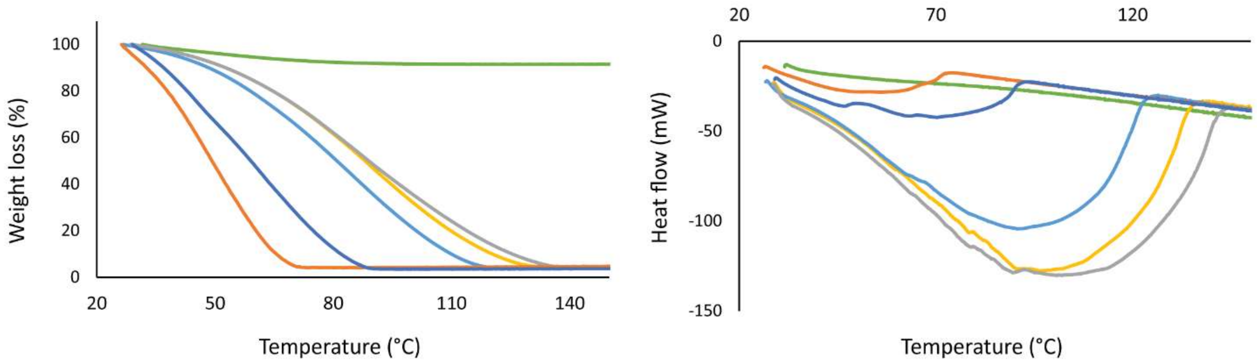

Figure S2: TGA analysis of the different chitin fibers. (top) The weight loss and (bottom) heat flow associated to the dehydration events are reported. The samples analyzed are: (green) the initial chitin, (yellow) 20G, (light blue) 22G, (grey) 22G con, (orange) 25G, and (blue) 27G.

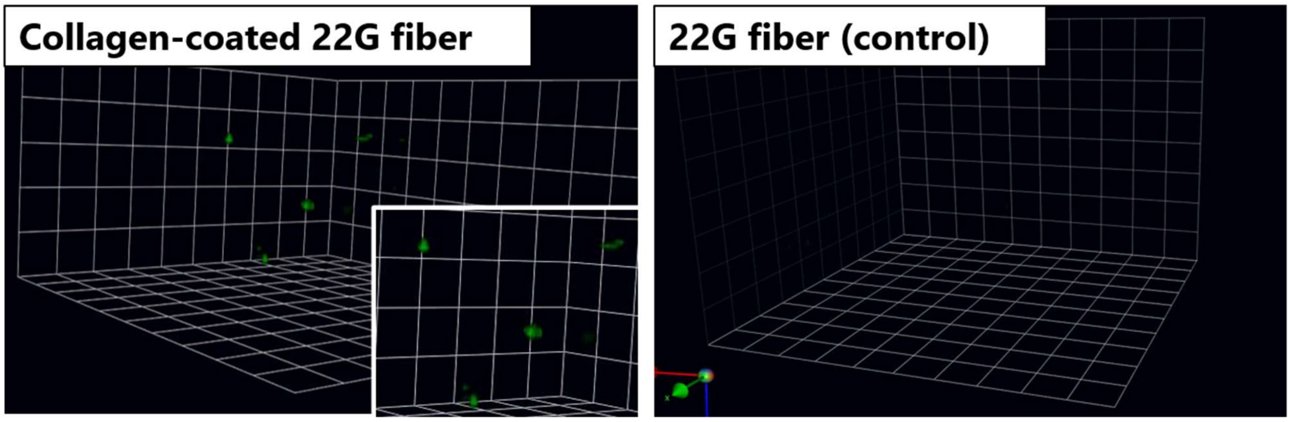

Figure S3: Confocal images of cells adhering to the chitinous scaffold. A confocal analysis of a chitin gel fiber with (left) and without (right) a collagen coating after being exposed to 1.0 × 105 cells for 24 h. An insight shows few cells adhering to the collagen-coated scaffold. Living cells were tagged using CellTracker Green.
